# Making More Sustainable Food Choices One Meal at a Time: Psychological and Practical Aspects of Meat Reduction and Substitution

**DOI:** 10.3390/foods11091182

**Published:** 2022-04-19

**Authors:** Elizabeth S. Collier, Anne Normann, Kathryn L. Harris, Lisa-Maria Oberrauter, Penny Bergman

**Affiliations:** 1 Perception & Design Unit, Division Bioeconomy & Health, RISE Research Institutes of Sweden, 114 28 Stockholm, Sweden; kathryn.harris@ri.se; 2 Department of Health, Medicine and Caring Sciences, Division of Society and Health, Linköping University, 580 02 Linkoping, Sweden; 3 Perception & Design Unit, Division Bioeconomy & Health, RISE Research Institutes of Sweden, 412 76 Gothenburg, Sweden; anne.normann@ri.se (A.N.); lisa-maria.oberrauter@ri.se (L.-M.O.); penny.bergman@ri.se (P.B.)

**Keywords:** climate change, consumer behaviour, meat substitutes, meat paradox, cooking at home

## Abstract

Switching out meat in favour of plant-based alternatives such as meat substitutes is an important step towards eating more sustainably. Here, the aim was to identify and explore the specific barriers experienced by Swedish consumers when replacing meat with more sustainable alternatives. All meat-eating participants in this study reported some interest in reducing their meat consumption. Aspects of home-use and central-location test methods were combined by using a digital conferencing system to host cooking sessions and focus group discussions online, which was shown to be a viable setup even in this hands-on setting. The discussions targeted participants’ experience preparing meals using meat substitutes as well as their perceived motivators and barriers to reducing meat consumption. Four themes identified through thematic analysis indicated that meat-eating participants, despite their desire or intent to reduce their meat consumption, experienced barriers relating to the following: internal conflict due to holding multiple positive and negative beliefs about meat simultaneously (*ambivalence*), justification of eating meat (*rationalisation*), a desire for variety in and control over their food choices (*agency*), and sensitivity to the views and expectations of other people and the situational context regarding meat (*social and structural* *factors*). Possible strategies to support ambivalent individuals in aligning their behaviour with their beliefs instead of vice versa are discussed in the context of the meat paradox. Agency and practical skills, including increasing knowledge in preparing meals with plant-based proteins, likely play a role in bridging this intention–behaviour gap.

## 1. Introduction

According to the most recent climate update report by the Intergovernmental Panel on Climate Change (IPCC), human influence has unequivocally led to widespread environmental changes. Achieving net zero CO_2_ emissions is now necessary in order to stabilise temperature increases. Importantly, within the agriculture and waste sectors (both significant contributors to these changes) livestock production is the largest source of emissions [1]. Thus, despite there being considerable variation both across food products and between producers of the same products [2], the IPCC report adds to the mounting evidence that meat production has an especially negative impact on the environment [2,3]. Drastic reductions in consumption of animal products at the consumer level are key to meeting global climate change mitigation goals [4,5]. Moreover, environmental impact is not the only concern that has been raised with animal husbandry and the demand for meat [6]. This is especially the case with confined animal feeding operations (so-called factory farms), which are associated with increasing antibiotic resistance, transmission of zoonotic diseases, and water contamination, as well as exemplifying animal cruelty at the hands of humans [7,8,9]. Thus, one way for consumers to contribute to a more sustainable food system would be a reduction in, or rejection of, meat products.

Despite being arguably one of the most powerful arguments against continued consumption of meat at current levels, environmental concerns and climate change have not always been among the primary reasons that people reduce or stop eating meat. In fact, the relationship between meat consumption and the environment is not necessarily well known among meat-eating individuals [10]. However, the Swedish Board of Agriculture does ascribe the gradual reductions in meat consumption since 2016 in Sweden in part to increased awareness of the environmental impact of meat among consumers [11]. Concerns around animal welfare and health tend to be stronger motivators [10,12]. The relative importance of these motivating factors (environmental concerns, animal welfare, health, etc.) also varies, for example, between flexitarians and unrestricted omnivores [13] and vegetarians and pescatarians [14]. Even the meaning of the term “health” as it pertains to eating meat (or not) is interpreted in very diverse ways, where variation can exist even within a single person’s experience [15].

Vegetarian and vegan diets are also sometimes assumed to be more expensive than a standard omnivore diet, making price another perceived barrier to sustainable dietary choices [16]. Behavioural economics studies have revealed that meat preferring individuals may be more likely to choose a plant-based burger only when the price was two-thirds or less than that of a meat burger [17], and that although consumers show some willingness to pay extra for meat products labelled as more climate friendly, they were willing to pay an even higher premium for meat products labelled as healthier or having better animal care credentials [18].

Even given awareness of the arguments for reducing their meat consumption, people remain resistant to behavioural change. Instead, many people find themselves facing what is known as the “meat paradox” [9,19,20], whereby morally conflicted meat eaters feel distressed that their behaviour is harmful but continue to eat meat. Explanations of the meat paradox are grounded in dissonance theory, which posits that inconsistencies between beliefs and behaviour create negative arousal and the state of cognitive dissonance. This in turn motivates mental strategies to reduce this negative arousal [21]. Resolving the meat paradox, thus, either requires that individuals bring their behaviour in line with their beliefs, i.e., by not eating meat, or that they bring their beliefs in line with their behaviour, e.g., by engaging in moral disengagement or avoidant strategies [9,19,22]. By providing justifications perceived as reasonable for a behaviour under scrutiny, rationalisation facilitates maintenance of a positive self-image [23] and continuation of behaviours or beliefs one may otherwise feel guilt towards [20,24].

Consumers sometimes conflate arguments for reduction with being asked to completely eliminate meat from their diet, which, in turn, can be linked to resistance to change [25]. It has been reported that people fear experiencing social stigma if they were to completely eschew meat [26], which may contribute to this resistance. This seems consistent with findings indicating that, at least among certain groups, communicating a message of reduction may be more effective than one of elimination [27]. That this effect was not ubiquitous across different groups may not be surprising given that motives for meat consumption are also affected by several demographic and socio-economic factors [28]. For example, there is an established relationship between masculinity and meat consumption [29,30]. In one qualitative study, the concept of masculinity was brought up in 100% of the focus groups, and the resistance of ‘husbands’ in particular was cited as a reason that a whole family did not eat meat-free meals more frequently [31]. Unsurprisingly then, given the complexity of the issue, willingness to reduce meat consumption is not shared by all consumers, and the number of meat-eating individuals who spontaneously switch to a vegetarian or vegan diet remains fairly low [32].

In addition to the barriers discussed above, sensory enjoyment of meat and uncertainty in how to replace it in meals also contribute to resistance to change [25,31,33]. Positioned in the market as direct replacements for meat products in meal applications, meat substitutes could offer consumers interested in reducing their meat consumption a route to change because these products may facilitate incremental reduction of meat [34]. However, these products are not always accepted by consumers as viable alternatives to meat, typically due to being perceived as sensorially inferior, expensive, and less versatile than meat [35,36]. Some consumers do not believe that it is necessary to explicitly replace meat in a meat-free meal [35], and they may even perceive meat substitutes as less environmentally friendly than meat [37]. The perception of meat as the main component or de facto choice in meals can also inhibit them from considering alternatives such as meat substitutes [38]. In their review of emerging themes in meat and milk alternatives research, Lonkila and Kaljonen [39] described these alternatives as hanging in an “inescapable tension” with their animal-derived counterparts, hoping to remove themselves from the negative impacts of meat and milk, while simultaneously aiming to replicate or replace their desirable aspects. Indeed, many meat substitutes intentionally attempt to mimic the taste and look of meat, which can be both an advantage and a disadvantage.

Acceptance of meat substitutes is also affected by the meal context they are served in, and these products are often perceived as less appropriate in certain meal situations (e.g., eating with family and friends) than either meat or other alternatives to meat, such as legumes and nuts [40]. Given that these products are often marketed as direct replacements for meat, consumers may tend to directly compare them with meat. This in turn may be detrimental to their acceptance because consumers’ sensory expectations of meat could lead to disappointment with analogue products [40]. It has been suggested that meat substitutes that retain key similarities with meat in terms of taste, texture, and ease of preparation may have the highest chance of success [41]. However, maintaining similarity with meat may not be, in itself, sufficient. Sensory expectations and preferences regarding meat substitutes differ between omnivores, vegetarians, and vegans [42], and meat mimicry can be perceived as unpleasant by some consumers [25]. Nonetheless, in order to be accepted as true replacements for meat, meat substitutes need to not only be sensorially enjoyable, but should also fulfil a similar function in meals and be perceived as appropriate to use across different meal and social situations. The pressure on meat substitutes to impress and provide a satisfying and tasty alternative to meat only increases when considering evidence that people who reject a product can take a long time before trying it again [43].

Here, we aimed to better understand people’s experiences of replacing meat with meat substitutes, by identifying both psychological and practical barriers associated with substituting meat. To this end, participants were invited to cook two dishes including meat substitutes with guidance from a professional chef and subsequently took part in focus group discussions online. Because the sessions were held online, participants were able to use their own cooking instruments in a familiar and comfortable environment, which was thought to improve ecological validity. Preparing and tasting dishes with meat substitutes was intended to enable them to be more specific in their discussion of sensory characteristics of such products [35] as well as aid identification of positive and negative aspects of preparing and handling such products both in isolation and relative to meat. Cooking in real time was also expected to increase participants’ sensitivity to specific obstacles they experience while preparing and cooking meals without meat, which has previously been identified as a barrier to reducing meat consumption [44]. A unique feature of this design was that by guiding participants through the meal preparation and exposing them to potentially novel recipes and products, we were able to directly tackle a common barrier to reducing meat consumption: perceived difficulty making tasty meat-free meals. This meant that specific practical concerns could be highlighted in real time, and that participants could reinforce their skills in meat-free meal preparation.

Finally, we primarily focused on individuals who already had at least some intention of reducing their meat consumption—but still experience certain hindrances—in an attempt to identify factors that inhibit fully implementing the changes they want. In this way, we aimed to understand the intention-behaviour gap regarding meat reduction. Furthermore, given that meat substitutes are sometimes described as transition products, this group may be expected to include those for whom these products are the most useful. Exploring the perceived positives and negatives of meat substitutes from conceptual to practical aspects among these individuals may help understand their role in effecting or inhibiting dietary change. We also included non-meat-eating participants, who may provide valuable insight into the potential of such products for maintaining change, as well as highlight the key motivators that led them to eliminate meat from their diet.

## 2. Materials and Methods

### 2.1. Participants

Prospective participants registered their interest through an online recruitment system widely used by researchers based in Sweden (Accindi). This system can be used to notify individuals registered as interested in study participation in the system database of the study via email. The advertisement specified that we were interested in individuals intending to reduce their meat consumption or who were vegetarian/vegan. The study was advertised only to individuals living in the Gothenburg/Västra Götaland region of Sweden due the practical need to collect ingredients for the cooking sessions from a specific location in this area.

Individuals who indicated interest in taking part were contacted by one of the researchers via email. The email included a link to a short screening survey requesting demographic information, the frequency they prepare vegetarian meals, the extent to which they agree that they care about what they eat in terms of themselves, the environment, and/or animals, as well as their availability to take part in the study on the dates it was planned to be run. Ninety-one individuals signed up and completed the screening survey. Individuals who stated that they never or very seldomly (<1 time/fortnight) prepared vegetarian meals (N = 6) and those who were unable to take part on the dates the study was scheduled (N = 10) were excluded. Of the remaining 75 individuals, 39 collected the ingredients at the designated location and took part in one online session. None of the researchers had any previously existing relationship with any of the participants, whose demographics are provided in Table 1. Participation was compensated with a gift card worth 400 SEK.

#### Data Analysis

The recorded focus group discussions after each meal were transcribed offline by A.N., E.S.C., K.L.H., and P.B. Thematic analysis [45] was used to investigate the transcribed data, using the RQDA package in R (version 3.6.3, The R Foundation for Statistical Computing). The transcripts were read and re-read by three coders (A.N., E.S.C., and K.L.H.), who independently made initial notes and sets of codes. Where relevant, aspects such as laughter and pauses were also noted, as well as how the participants interacted with each other, e.g., if they interrupted others. The initial codes were discussed among the coders, inter-coder discrepancies were discussed and resolved, and a common codebook was generated. To relate these codes to the research question, they were broadly categorised under positive and negative aspects of meat and/or meat substitutes. During a second round of independent coding, additional codes were identified, whilst others were renamed or excluded by the coders. These were discussed further and grouped into initial conceptual themes and subthemes based both on the (interpreted) semantic content of the coded statements, the context in which those statements were made, and with relation to the previous categories of negatives and positives about meat and meat substitutes. Further discussions and re-grouping of codes were held until the first theme structure was generated. Two further rounds of discussion and engagement with the material were held, after which the final theme structure was created.

In the interests of transparency and acknowledging their active role in analysing the qualitative data [45], the authors would like to disclose the breakdown of dietary choices within the research group and provide a brief description of the societal context in which the work was conducted. The authors consist of one vegan, one pescatarian, and three flexitarians. The authors are all white women who currently live and work in a Scandinavian/European cultural context, specifically in Sweden, where environmental issues are regularly discussed in the media and increased sustainability in food systems has been highlighted as a key issue by the Government in the first National Food Strategy for Sweden [46].

## 3. Results

**Figure 1:**
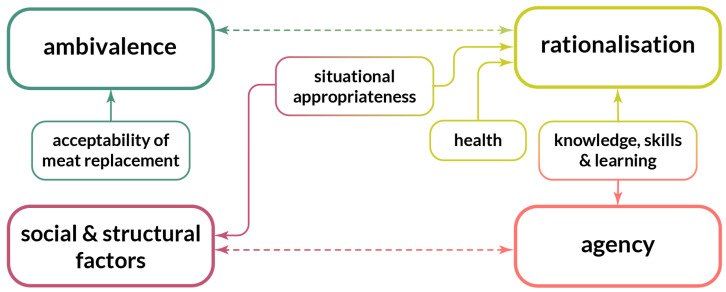
The four themes, and their associated subthemes, identified through thematic analysis.

Four themes were identified: *ambivalence*, *rationalisation*, *agency*, and *social and structural factors,* see Figure 1. The themes are explored in the sections that follow, and anonymised quotes along with relevant demographics are provided to illustrate the themes and their subthemes. The speakers are denoted as gender-age-frequency of cooking vegetarian meals (according to the letters shown in Table 1), e.g., F29-b would refer to a 29-year-old female who prepares vegetarian meals 5–7 times/week, and M60-e would refer to a 60-year-old male who prepares vegetarian meals one time/week.

### 3.1. Theme 1: Ambivalence

Ambivalence was prevalent among the majority of the meat-eating participants across the sessions. Conflict manifesting from balancing numerous thoughts regarding enjoying meat, protecting the environment, animal welfare, and health seemed, in some cases, quite extreme, with one person stating that:

“*Sometimes it can take many hours for me to do the grocery shopping, and then you get a bit tired of yourself. It’s quite hard to be aware.*”

(F36-d)

Many meat-eating participants indicated that they were strongly considering eating less or rejecting meat entirely, but that they simultaneously did not feel able, willing, or ready to do so. Meat-eating participants often expressed disgust with the meat industry, and distress at thinking about animal suffering. Some even bluntly stated that they did not want to contribute to this system, but admitted that they continue to eat meat anyway, sometimes redirecting the conversation away from animal welfare. Such redirections, typically to health or practical concerns (e.g., difficulty accessing alternatives to meat or vegetarian recipes) seemed to serve as a strategy to explain why they struggled with changing their behaviour, despite their awareness of the negative aspects of meat consumption. These were not justifications of current behaviour per se; rather, participants revealed the hinders and conflicts they perceived between their beliefs and their behaviour, as exemplified through this interaction:

F24-f: “*I try to eat a lot of vegetarian food because I struggle with the animals, how they are treated.*”

Interviewer: “*Ah, it’s an important aspect for you?*”

F24-f: “*Yeah, it’s an important aspect for me, but then it’s also, you need to think about nutrition too, not everything that is vegetarian is nutritious either. I’m not vegetarian, like I eat everything, but I’ve appreciated that these alternatives exist more and more, when they have good nutritional content too.*”

The extent to which cost generated ambivalence varied considerably across participants. For some, price was perceived as a barrier to reducing their meat consumption. Others argued that it was cheaper to avoid meat because meat itself is expensive. Notably, there was sometimes a lack of nuance when participants gave their opinion on price affecting their food choices, with participants often stating bluntly that they thought that meat or vegetarian options were cheaper without elaboration. It was, however, pointed out that the relevance of price to food choice is affected by a person’s life circumstances, and that the extent to which price affects continued meat consumption likely varies with socio-economic status. Others said that price may not be a barrier for them if they were testing new products once, but it may become more relevant if a product is intended as a longer-term staple in their diet, and some were willing to pay more if they were confident the ingredients were high quality.

“*I agree that if you’re testing something [the price] doesn’t matter, but if it were to be something you get really into so it could be for everyday consumption, like two times a week if it’s something good, then maybe it, maybe not a go-or-no, but you can reflect about the price, I think.*”

(F35-d)

Some participants expressed that although they thought meat substitutes were expensive, not all alternatives to meat were pricy (beans were often named as a cheap alternative) and some also mentioned that meat substitutes are often on offer at supermarkets. Thus, although price was a source of conflict for some participants, there was no consensus among participants regarding whether they perceived a vegetarian/vegan or omnivore diet as more cost-effective.

“*Like, for my part maybe I don’t need to care about whether it costs more or less, but I do anyway, I feel very aware of it.*”

(M61-e)

As might be expected, ambivalence was less, or not at all, prevalent among participants who do not eat meat, who instead were clear in their motivations for eliminating meat from their diet. Typically, their reasons were similar to the concerns provided by meat-eating participants for intending to or having already reduced the amount of meat they eat, namely animal welfare, health, and environmental reasons. Animal welfare seemed to be a strong motivator among these participants, with other aspects such as the environment and health perspectives being perceived as additional benefits.

“*Like, animal handling is absolutely the most important [aspect] for me, and then the other factors are strong too. Yeah, animal handling is the most important and then the others are good bonuses.*”

(F21-a)

“*No, it was the animal industry and it, like in the beginning, then there’s been more and more discussion about the environment in the past fifteen years so now it’s because of the environment.*”

(F36-a)

A subtheme regarding the acceptability of meat replacements emerged here.

#### Acceptability of Meat Replacement

Some felt like they should cut down their meat consumption but held negative perceptions or associations with candidate replacements for meat. Negative views on meat substitutes as adequate replacements for meat materialised, and it seemed that some participants held double standards regarding the expected sensory properties and acceptability of meat substitutes relative to meat. For example, some participants stated that they tend to read the ingredients list of meat substitutes but not of processed meat products. Participants placed high expectations on meat substitutes regarding their capacity as a replacement for meat, which, when not met, seemed to land them back in an ambivalent state due to the conflict not being resolved.

“*Yes, it should be healthy, so that there aren’t additives that aren’t good for you, like it shouldn’t be too chemical or processed in any way, but anyway they should be, uh, good products.*”

(F49-e)

Meat substitutes were compared both favourably and unfavourably with meat, and participants also discussed their sensory experiences of different meat substitute products. Some participants expected lacklustre texture and inferior taste from meat substitutes. On top of feeling that meat substitutes in general do not typically taste as good as meat itself, a number of participants mentioned experiencing an after-taste—that they found difficult to describe but did not like—when they had tasted meat substitutes in the past. Of the two products tasted in the cooking session, the pre-flavoured product was typically preferred by these participants since the aftertaste was less obvious. These poor sensory reflections were not always sufficient to be considered problematic, and the majority of participants mentioned at least one meat substitute product that they had positive views of. For several participants, vegetarian dishes and meat substitutes were just as sensorially pleasurable as meat, although it was typically noted that they still find meat enjoyable. These positive reactions to the taste and texture of meat substitutes, including the products used here, were often in the context of the product resembling its meat analogue. On the other hand, some participants were willing to accept sensory deficiencies if the products came with other benefits, such as a lower environmental impact.

“*Nah but I thought the texture was much more meat-like than, like, I like meat so it was more comfortable, like as a substitute for meat.*”

(F19-e)

“*On the other hand, I know that they have several difficulties often because there are challenges with texture and taste and so on but in some way I feel like I accept that if they have other advantages like lower emissions and maybe they’re richer in fibre and such, healthy.*”

(F35-d)

Reactions to the appearance, taste, and texture of the products used in the cooking sessions were typically highly positive, sometimes to the surprise of the participants themselves. While many participants mentioned the wide variety of meat substitute products available on the Swedish market, others stated that they did not think the range was very big, and gaps in the market were identified and discussed. Many felt that there was a lack of substitutes for fish products. Notably, they often indicated that they may not use substitutes for fish products even if they were available because fish and seafood in general was perceived as too good to be replaced. This exemplifies the dashed line drawn between ambivalence and rationalisation (Figure 1), since the perceived lack of substitutes for fish was identified as a barrier to replacing it (“can’t change”), but the defensive rationalisation that it cannot be replaced anyway because it is too tasty (“won’t change”) seemed employed to avoid the discomfort of thinking about rejecting fish even if substitutes were more available.

“*There are lots of substitutes for meat but nothing for fish and shellfish, and fish and shellfish are actually very good, I think. So, if there’s nothing that can replace it, I would find it very difficult to be a vegetarian completely. I can maybe skip the meat and replace it with vegetarian products, but there is still the rest, that from the sea. I don’t want [to replace] that.*”

(F57-e)

It also emerged that not all meat products were perceived as equally replaceable. For example, reducing meat consumption by replacing items such as meatballs or mince seemed fairly trivial for many, but other products such as steak, chicken fillets, or fish were perceived as harder to replace or reject. In some cases, these were perceived as not having a suitable alternative and, in others, participants simply liked the taste and did not wish to miss out. This was also the case for specific dishes, with dishes where participants perceived most of the flavour coming from spicing and sauces rather than from the meat itself were judged to be those where meat was more easily replaceable. Such perceived problems with candidate alternatives to meat seemed to generate conflict about reducing or eliminating meat consumption among participants.

“*No, but I know myself that it doesn’t sound logical. I don’t understand why I can eat a piece of meat where it’s very clear that it’s meat but on the other hand I can’t eat meatballs which I could trick myself into thinking were vegetarian since they look the same. But it doesn’t work.*”

(F29-b)

### 3.2. Theme 2: Rationalisation

Rationalisation or justification of meat consumption was fairly common among the meat-eating participants. Many, even those who were excited about or positive towards the increased variety of meat substitutes available, believed that replacing meat was difficult or that they did not wish to completely abandon meat. Some participants claimed that they had already reduced their meat consumption or that they do not typically eat very much meat anyway and so do not feel they need to reduce further. It was also argued that meat is necessary for a satisfying meal, and some participants expressed not feeling full after eating a dish without meat. Perceived negative aspects of meat substitutes were also provided as justifications for not using them as replacements for meat. For instance, concerns around the environmental impact of long-haul transport and the destruction of the rainforest associated with the production of soy led to even participants who do not eat meat to suggest that just because a product is vegetarian does not necessitate that it is always a better choice. The products used in the cooking sessions were based primarily on oats. For some, the process of obtaining a “chemical product” from raw materials such as oats was perceived negatively, in which case other alternatives to meat, such as chickpeas, were sometimes viewed more favourably. On the other hand, oats were also described as a more “Scandinavian” raw material which could be grown in Sweden, and this was generally received positively by participants, especially among those concerned about reducing impact on the environment.

Along similar lines, choosing Swedish meat was perceived as less environmentally and ethically questionable, and participants justified their meat consumption by pointing out that they only chose Swedish produced products to the best of their ability. Rationalisation around animal welfare emerged when participants discussed their disdain for the meat industry but qualified their own meat consumption by stating they choose higher quality meat or meat that they or someone they knew had hunted themselves, which was perceived as less ethically problematic. Participants also discussed the perceived compromise between animal ethics and meat consumption by eating what they perceived as good quality meat, less often.

“*Because I want to know that the animals have been raised in a good way, treated in a good way, I believe that we in Sweden have a good framework compared to some other countries, with how animals are treated and such, and you want to know which farm it comes from and such, and my husband hunts so we eat game and then you know that they’ve had it good, they have ran around in the mountains like, such things I think are more important than buying the cheapest. Then you can eat meat less often and eat good meat, like.*”

(F51-c)

Within rationalisation, subthemes of (i) situational appropriateness, (ii) health, and (iii) knowledge, skills, and learning were identified.

#### 3.2.1. Situational Appropriateness

Some participants reasoned that meat consumption was justified depending on the situation, and a repeated example of this related to grilling meat at summer barbeques, where no suitable alternative was perceived to exist by some. For others, grilling represented a situation where meat was justified because of tradition and expected taste. There was a perceived lack of vegetarian options in restaurants as well, making this another situation where participants found it reasonable to choose a meat or fish option instead. One participant mentioned that although they reject eating shellfish at home, they do still eat it at restaurants, highlighting the influence of the situation on food choice. Choosing meat was also justified at other holidays and special occasions, with many meat-eating participants reasoning that although vegetarian food may be appropriate as an everyday option, it lacks status and a feeling of luxury. As such, meat was seen as more fitting for weekends and special occasions such as New Year’s Eve in a way unmatched by vegetarian options.

“*Yeah of course it is somewhat tradition, but it’s like, if you’ve tried to think like ah but we won’t take that [meat] we’ll take something vegetarian, what do we take then? And then there isn’t anything that feels, like, that has the same status.*”

(M32-c)

“*At the weekend it will maybe be meat or fish or, because those I see maybe as more festive in some way.*”

(F49-e)

#### 3.2.2. Health

The participants valued their health and appreciated the connection between their diet and their physical health. For a number of participants, this was a motivating factor for reducing their meat consumption, in particular of red meat, as exemplified by a participant stating that their decision to reduce meat is:

“*…not only about the environment, it’s simply myself, like egotistically motivated, that I want to be healthier.*”

(F52-c)

On the other hand, health was a rationalisation for *not* reducing or eliminating meat from the diet for many participants, who instead valued what they described as a mixed or balanced diet. Concerns around obtaining sufficient nutrition, both in terms of macro- (e.g., protein) and micronutrients (e.g., iron, Omega 3 fatty acids, vitamin B12) on a low- or no-meat diet were voiced, and in some cases stated as justifications for not deviating too far from their current eating habits. Others believed that they did not need to keep track of the nutritional content of their meals because they eat a mixed diet, whilst vegetarians and vegans may need to be more conscious of what they consume in order to stay healthy. Some also expressed a dislike of the over-processed nature of meat substitutes. This seemed to justify for participants both that meat is necessary for health and that eating it mitigates the need to worry about nutritional deficits. However, it was also discussed that people probably get enough protein regardless of their dietary choices, and it is unlikely that reducing or eliminating meat would be problematic on this basis. Regarding meat substitutes, there were some concerns regarding their nutritional quality, and whether or not these products provide similar nutrition to meat generated scepticism to their use.

“*I think that there is a lot or there has been a lot of flour in these substitutes and a lot of sugar and such. I think it’s strange that they, because anyway you maybe think that they’re a healthier alternative, but it could be that they pile on other bad things then.*”

(F40-b)

#### 3.2.3. Knowledge, Skills, and Learning

A perceived lack of knowledge and skills among some participants in choosing, preparing, and serving vegetarian ingredients (including meat substitutes), as well as not wanting to expend time and effort finding out, was a reason for not having reduced or intending to reduce meat consumption among some participants. It was discussed that the cooking sessions were valuable because participants received guidance and could ask questions about the product and how best to handle and store it, and some indicated that they would have had more difficulty if they had not received instruction. It was also suggested that they would be more likely to use meat substitutes if supermarkets offered recipe suggestions or try-before-you-buy opportunities in-store. The inclusion of recipe tips on packaging was also suggested. For some participants, inadequate knowledge of the pros and cons of different substitute products (e.g., regarding their raw materials such as oats vs soy) or not knowing where to find these products in supermarkets were given as justifications for not using them more often. It was suggested by some meat-eating participants that meat substitutes should be located in the same aisle as meat in supermarkets, in order to make it easier to find them and highlight that they exist as an alternative.

“*It feels like you don’t really know, that you maybe don’t want to bother to find out either, so if you had known that this soy mince, that it was just as good as this amount of beef mince, then I would have managed it.*”

(F23-d)

### 3.3. Theme 3: Agency

While tasting the dishes, it was common that participants discussed ways that they would personalise or change the recipe to better fit their own tastes, implying a desire to make their own mark during food preparation. Many participants seemed keen to retain control over what foods they ate. This was partly evident through assertions that meat substitutes seem to have “strange” and “weird” ingredients that participants were sceptical of and unfamiliar with, making them an undesirable choice, and through the stated preference of making vegetarian alternatives from scratch instead of purchasing them ready-made. It was, however, noted by some participants that one did not necessarily want to know what was in the meat products they bought, or where they came from, either. Maintaining agency also emerged in the fact that many of the participants were not willing to completely cut out meat, instead preferring to at most reduce or eliminate red meat while retaining other meat and seafood products in their diets.

“*It works sometimes, but I wouldn’t feel good having it as a steady diet, I need the meat, fish, chicken, then my family in general wouldn’t really become vegetarian so it’s about finding something in common. But I like, and we like, to make vegetarian dishes too, so we mix it up.*”

(F55-d)

Aside from enjoying the taste, participant’s desire to continue eating these foods was associated with their belief that they should not have to sacrifice their own health by reducing or eliminating meat from their diets. For some participants, then, agency over their food consumption was directly associated with good health, which in some cases led to resistance to drastic changes in their current habits. For others, this was motivation for having already reduced their meat intake, though they made it clear that elimination was not an option for them as it would mean loss of control over the nutrition.

“*I think more like, I don’t know, it maybe isn’t so healthy [to only eat meat]. So, I believe in a mix, like. I don’t want to go over to only vegetarian or only eat something else, it should be a mix of both meat and chicken and vegetarian, so that you get everything somehow.*”

(F49-e)

Some participants expressed wanting to eat meat simply because they enjoy it. In such cases, the idea that discussions around meat consumption were polarising occasionally arose, where some participants described feeling as though they were being judged for their choice to eat meat. For example, one participant defended their intention to continue eating meat at their current—nominally low—level, describing their scepticism that trying to change consumption habits at a large scale could work, and that for some people, cutting it out completely would be impossible:

“*It is often not one or the other, like not everyone can eat meat, no, but not everyone can stop either.*”

(F56-d)

Another participant (F21-a) who had recently become vegetarian noted that they had attended many dinners where the vegetarian options were lacking. The prospect of having fewer food options available if they cut out meat entirely, both in general and in specific situations, e.g., when eating out, was unappealing to many participants, many of whom valued variety and choice.

“*Not really, but you think that a piece of meat, or fish, if you’re vegetarian then there’s no shrimp, nothing. I mean, I find it hard to skip shellfish. I don’t want to.*”

(F57-e)

The subtheme of knowledge, skills, and learning emerged here as well as within rationalisation.

#### Knowledge, Skills, and Learning

Having knowledge of and enjoying cooking seemed associated with a desire for feeling like one is actually making something, which some participants perceived as a drawback of meat substitutes, seeing them instead as a shortcut or a lazy alternative to “real cooking”. For some there was a preference for spicing and creating dishes on their own terms and being in control of the raw ingredients used—applying both to meat and vegetarian dishes. This preference for control over the cooking process translated often to a preference for using the meat substitute that was unflavoured (that they could flavour themselves) in the cooking session, rather than the pre-flavoured product (where the flavour was pre-determined).

“*I want to feel like I’m cooking. Even if I buy something semi-manufactured or whatever you want to call it, I still want to have the feeling that I’m cooking.*”

(F36-d)

“*Yeah, because if I buy them ready, these steaks, they’re not always good, I can’t have it that way, I would like to put my own stamp on it, if I were to have something like this [unflavoured product] I could spice it and know what’s in it.*”

(F51-c)

For other participants, however, the flavoured product was appealing because of its ease of use, convenience, and lower demand for skills and effort to prepare. Learning about vegetarian cooking and trying new recipes, for example through vegetarian cookbooks, seemed to be methods by which meat-eating participants could take control and change their cooking and eating behaviour. In a similar vein, one vegetarian participant noted that their knowledge of and skills in cooking had grown since giving up meat:

“*I think it’s more like I make much more food, and more recipes and more from all the corners of the world, so I think I’ve only broadened what I make.*”

(F21-a)

Regarding the role of meat substitutes, for some participants it was important that these are easy to use in already-familiar recipes, and others suggested there is value in being able to trick themselves and others using these products. It was discussed that the cooking methods used in the cooking session were familiar, which was seen as a positive. It was also discussed that making vegetarian food does not have to be as difficult as people assume. It was suggested by some that meat substitutes might be useful as a bridge for learning about cooking meals without meat, as these could be incorporated into the person’s diet without necessarily demanding additional skills and effort. This view was not shared universally, however, as others perceived entirely new recipes and flavours as more advantageous to facilitating habit change due to not inducing a direct comparison with the original meat-based recipe.

“*It’s one way of learning the cooking and such, it can be a step, like the beginning and so you take dishes you recognise and switch things out.*”

(F59-d)

“*I think it’s easiest to switch to something completely new because otherwise you’re a little set in the old track, you think, ah but it should taste like this.*”

(F38-b)

### 3.4. Theme 4: Social and Structural Factors

There were a range of external (social and structural) factors that affected participants’ choices regarding meat. A major factor was the influence of other people, such as family, friends, and co-workers, who provided both supported and hindered reducing meat consumption.

“*The best thing about meat? I don’t think there’s much that’s the best about meat. I probably wouldn’t eat meat if my partner didn’t eat meat actually.*”

(F27-b)

“*Yeah, I have a lot of vegetarian friends at work, and vegans, so I get a lot of tips from them. What I should buy and shouldn’t buy, and what’s good and not good.*”

(F57-e)

The notion that it is generally easier to eat vegetarian dishes alone than in the company of others was shared by several participants. There was also discussion that eating less meat may have a generational component as it was perceived as something more appealing to younger than older people. The influence of other people on people’s food choices was evident in discussions on being exposed to meat substitutes and vegetarian alternatives by family members, friends, or colleagues, having others in their social circles who continued to eat meat, and that in the absence of other people it is easier to opt for vegetarian dishes. This also exemplifies how agency and social factors do not always align and can instead lead to tensions in meat-related food choices (represented with a dotted line between Agency and Social and Structural factors in Figure 1).

“*If I were to cook for someone else or for the family, then I might eat more [meat] but if I’m alone then I eat nearly only vegetarian, basically.*”

(F40-b)

Through participants descriptions of the anticipated reactions to reducing meat from other people they interact with, it seemed as though at least some may also be subject to ambivalence similar to that which emerged from the participant’s own experiences. The expectations of other people regarding food were perceived as important, and some participants believed that meat or fish dishes were expected at dinner parties while vegetarian food might subvert expectations. That meat substitutes should resemble their meat equivalents in texture and taste was perceived as even more important when cooking for other people than when choosing products or preparing meals for oneself.

“*Yeah exactly, and then I come back a bit to that it needs to be accepted by many, so that when you eat alone it’s very easy but if you have that step to take, to convince someone else, then there is higher demand of the taste.*”

(F35-d)

A vegetarian participant (F21-a) mentioned enjoying the “whole culture” around being vegetarian and discussed the excitement that exists around experimenting with new alternatives to meat within this group. Exposure to information about the impact of meat on the environment through, for example, newspapers as well as discussions about these issues with others in their social circle also played a role in shaping people’s beliefs. Some also mentioned a need for rethinking typical eating patterns to protect the planet and others in society, that people cannot continue consuming meat at current rates, as well as the desire for finding suitable alternatives capable of facilitating normalisation of reducing meat.

“*That which was normal in the past, but not normal in the present and the future, and it isn’t going to be the same as when we were adults. It’s a bit of a home mission. To have a good alternative that you can accept and normalise.*”

(F35-d)

Another group who influenced food behaviour was the participants’ children; both those who did not eat meat (much or at all) and encouraged a similar change in their parents, and those who were not at all interested in rejecting meat. Some participants described having experience buying and using meat substitutes as a result of having children who had become vegetarian or flexitarian. Other mentioned that having children who ate a vegetarian or flexitarian diet helped them learn more about vegetarian cooking as they could find and prepare recipes together. As such, through children who had stopped eating meat, participants gained exposure to new products and cooking non-meat recipes, which influenced their attitudes regarding meat and its substitution:

F61-d: “*Yeah. But it’s my youngest son who has taught me there, so I haven’t come to it myself. It’s also a generation question, I wouldn’t have dared if he hadn’t gotten into it.*”

Interviewer: “*How did he do that? Did he say ‘mum, buy this’ or did he make something that you got to taste?*”

F61-d: “*Nah, he came over and we made it together, and we Googled how it should be done. Because he eats nearly exclusively vegetarian. For the environment and, or well it’s mostly the environment, I think.*”

Opinions were somewhat mixed regarding anticipated reactions to the products used in the cooking sessions from children and other family members, though most believed the dishes would be liked. Occasions of successfully tricking their children using convincing meat analogues in the past was mentioned, whilst others discussed the difficulty of using anything other than meat when children were at home due to the children’s preferences.

“*I also think that it is reminiscent of normal mince. I don’t think the kids would react much if we had this instead of meat.*”

(F30-e)

“*Then it’s that we have family dinners with the step-children, then they get to choose was we eat, but if I’m alone or with my partner we eat vegetarian as often as we can, like.*”

(F40-b)

The subtheme of situational appropriateness was shared with agency.

#### Situational Appropriateness

Eating with other people affected people’s food choices; for example, it was noted that although they may not prepare meat for themselves, they would eat it if it had been prepared for them:

“*So, when you’re in the shop, you should think about what is good for your body, and that is vegetables and beans and more natural products. But of course, I can eat red meat and chicken if it’s there but, uh that’s how it is.*”

(F59-d)

Example discussion topics included that hosting dinners for other people guides the selected menu and meat is typically expected and, thus, chosen, difficulty cooking meals without meat in a family setting, and the (in)appropriateness of trying to substitute meals in traditional family recipes. For non-meat-eating participants, re-inventing traditional dishes typically served at social occasions, for example Christmas and New Year’s Eve, as a family was a method to combat the perceived inappropriateness of alternatives to meat in these situations.

“*Nah but I think we’ve found our way to do it, and like Christmas food we’re really, in our family we make our own alternatives and our own balls with our own spicing and like, some salads and we do, ah but lots like that, we have really found our own way and our favourite dishes and that’s festive food for us in some way.*”

(F21-a)

The situation of eating a meal with other people and how food choices affect the mood and social dynamic was also discussed. Some participants described hosting a dinner that friends who do not eat meat would attend as an appropriate situation to cook a vegetarian meal and use meat substitutes in order to avoid having to make more than one dish. However, as exemplified in the exchange below, others were sensitive to perceived negative aspects of demanding more of their host by rejecting meat, as well as that they themselves may feel annoyed if someone they had invited for dinner demanded such additional effort from them:

F66-d: “*Yeah, if everyone else eats meat then maybe it’s a bit more awkward if you would eat vegetarian.*”

F25-b: “*Yeah, it would be.*”

Interviewer: “*Would you feel a bit annoying then? Why is it awkward?*”

F66-d: “*Well, then they need to, if they were, if everyone else is a meat eater and now they need to make something extra, then you feel awkward.*”

F25-b: “*And I think like when, if people have come over to my home and I need to make something extra because they don’t eat it, then I think that’s awkward.*”

## 4. Discussion

The aim of this study was to explore which obstacles individuals who intend or have already reduced their meat consumption face, and the role of meat substitutes in this. A virtual central location was created using conferencing software, where groups of participants prepared two meat substitute-based dishes and discussed their experiences of preparing and consuming meat and meat substitutes as well as their perspectives on meat consumption more broadly. The novelty of this study is both in its design where participants actively engaged with meat substitutes through guided meal preparation, allowing them to be specific in the obstacles that they experience (or not), and its focus on individuals who identified as intending to change their meat consumption and/or had already done so. This meant that barriers to *implementing* change, i.e., the intention-behaviour gap, became a stronger focus. Thematic analysis revealed several factors affecting choices and behaviour regarding meat and its substitution among these participants, primarily *ambivalence, rationalisation, agency,* and *extrinsic factors.*

Although all four themes are interrelated, they may be conceptually groupable into two pairs: the interplay between ambivalence and rationalisation may represent consumers’ internal difficulty in consistently making sustainable food choices, while agency and extrinsic factors seem to reflect the challenge in enforcing (or retaining a feeling of) their own choices and actions within social and societal contexts that do not reflect their motives or attitudes. Agency and extrinsic factors can also be complementary, e.g., encouragement from other people who have already reduced or rejected meat consumption can bolster one’s intention to do the same. These theme pairs are discussed in the sections that follow.

### 4.1. Ambivalence and Rationalisation

People who are ambivalent about meat are those who have mixed feelings towards it, i.e., they hold positive and negative feelings or beliefs simultaneously [47]. Here, ambivalence typically stemmed from holding negative views of meat in terms of environmental, ethical, or health consequences and positive views in terms of sensory enjoyment, social status and expectations, habit, and even health (which was both a motivator and a barrier to reducing meat consumption). Ambivalence regarding meat has been previously reported to moderate the relationship between attitudes towards and intention to follow a given diet (omnivore, meat avoidant, vegetarian, or vegan), such that attitudes more strongly predicted intention when ambivalence was low [48]. Ambivalence may be lowest before contemplating or after maintaining behavioural change, and higher during the intermittent stages [49]. Since we selected for these intermittent stages here, it is, thus, not surprising that we often observed a mismatch between the attitudes and behaviours of our meat-eating participants, while this was essentially absent among the non-meat eaters. Armitage et al. [49] further suggest that being in a state of ambivalence may promote openness to persuasive communication for these intermittent groups, as their information processing may be heightened. This is one reason why these people in particular may be of interest regarding the promotion of more sustainable dietary habits. Indeed, providing information about the climate impact of meat may primarily affect those who already believe meat consumption negatively impacts the environment [50].

It has been found by Berndsen and Van der Pligt [47] that more ambivalent omnivores associated meat consumption with risks for their health and the environment as well as negative affect, while Buttlar and Walther [51] found that ambivalence moderated the association that omnivores attribute animals as possessing minds and emotions to a lesser extent than non-meat-eaters. This moderation indicated that only meat-eaters with greater ambivalence attributed significantly less mind and emotional capability to animals. Such effects were not found for other rationalisations for meat consumption (based on the 4Ns, namely that meat consumption is ‘nice, natural, necessary, and normal’, see Piazza et al. [20]). While the negative impact of meat consumption on health is relatively demonstrable and salient, the mistreatment of animals and their possession of a mind may be a less tangible topic, which perhaps provides leeway for some individuals to construct a more comforting view of their choices. Speculatively, this may also be the case for climate change, where the magnitude of the issue may be difficult to fully comprehend, making it challenging for some to assess how individual action could matter in tackling such a sizeable issue.

The consequence for these ambivalent meat-eating individuals who show disdain towards the negative consequences of meat consumption yet continue to eat it, is typically what is defined as “the meat paradox” [9]. Indeed, we observed that those individuals who eat meat were eager to switch topics in order to avoid confronting what they perceived to be the particularly uncomfortable aspects of eating meat (e.g., avoiding continued discussion of animal welfare in favour of nutrition as exemplified in the results). It then follows that here the theme of rationalisation likely reflects participants resolving the meat paradox by justifying their behaviour with arguments based on other beliefs about meat eating. Such justifications included that meat is perceived as enjoyable, nutritious, socially expected, and normal. The subthemes that emerged within rationalisation (health, knowledge skills and learning, and situational appropriateness) seem qualitatively similar to previously reported justifications for resisting reduction or rejection of meat [20,33,52,53].

However, resolving the meat paradox by bringing one’s beliefs in line with one’s behaviour through rationalisation and moral disengagement strategies, as opposed to bringing one’s behaviour in line with one’s beliefs by eschewing meat, could lead to unintended consequences, such as reinforcement, risking invoking stronger commitment to current behaviour [22]. Investigating how to communicate information in ways which counteract moral disengagement rather than facilitate this reinforcement cycle, Buttlar et al. [54] suggested that a two-stage approach could be effective. In stage one of their studies, a person’s passive dissonance avoidance (strategies to circumvent the meat paradox in the first place, e.g., by mentally dissociating meat from the animal it comes from) was tackled by providing distressing information and images of the poor conditions and treatment of animals within the meat industry. Then, in stage two, moral disengagement was countered through dialogues regarding the person’s reservations and views on the issue.

Buttlar et al. [54] argued that this approach anticipates and accounts for rationalisation and disengagement by encouraging immediate counteraction of it through discussion and dialogue. Such an approach may be more effective in encouraging people to reduce or reject meat than providing information alone [54]. It has also been found that people were more receptive to anti-factory farm messages and more willing to decrease their meat consumption after being primed to adopt a stance on the issue [55]. Stance adoption was thought to increase experienced meat-related dissonance and motivate openness to aligning behaviour with belief instead of engaging in rationalisation. Such results are valuable as it remains unclear how to communicate information about meat consumption in ways that maximise effecting change. The focus of Buttlar et al. [54] was animal welfare and it would be interesting, for example, to evaluate whether a similar two-stage process would be effective in communicating other negative aspects of meat consumption, such as its environmental consequences.

### 4.2. Agency and Extrinsic Factors

Food consumption involves social aspects as well as individual motivations [28], and both context and the perceived expectations of others affect people’s impressions of when it is and is not acceptable to reject meat. For example, Elzerman et al. [40] found that meat was perceived as more appropriate to use than meat substitutes by Dutch consumers in many situations, including when eating with family and friends. People also tend to eat more meat when eating with family members than when eating alone or even with other companions such as friends or colleagues, as well as at the weekend, and while eating out e.g., at restaurants and cafes [56]. Additionally, non-vegans can experience fear of social ostracism or stigma from other people if they were to adopt a vegan diet [26]. The ways in which other people and situations affected the food choices of the participants in the present study were largely consistent with these earlier observations: for some reduction, but not elimination, was their goal, and many participants could give examples of situations where they did not feel comfortable or able to choose a meat-free option.

Speculatively, the influence of other people and social expectations on food choices even among those who wish to change may be partly driven by a preference for negative affect, a phenomenon that occurs in contexts where it is implied that some emotions are more appropriate than others [57]. Here, we observed that a desire for agency over one’s own food choices was a major motivator for food choice, until meals had a social aspect, when the concerns and expectations of family members or friends often took precedence. In other words, in the absence of social or familial context, people who want to reduce or reject meat tend to maximise positive affect by avoiding meat. However, the preference for negative affect phenomenon may drive them to instead opt for meat in many social contexts, possibly in order to avoid inducing negative affect in someone else, even at the expense of their own agency. This may, in turn, contribute to the ambivalence experienced by these individuals or lead them to rationalise that their behaviour was unavoidable in such a situation. Such a mechanism would likely work in tandem with other aspects, such as social pressure and fear of stigma, as discussed above. However, for those who intend to (further) reduce their meat consumption, we suggest that the preference for negative affect may act as another barrier to (continued) change.

Inversely, many participants also mentioned that exposure to and dialogue with individuals who do not eat meat was a motivator to reduce their meat consumption. Receiving recipes from meat eschewing friends or colleagues was mentioned as a valuable source of inspiration for exploring meals without meat. Although some mentioned that planning social events including food with non-meat eaters in mind may be awkward, for others this was a clear path to increased exposure to these products (e.g., preparing meals for visiting family members including meat substitutes). This role of meat substitutes as a way of being inclusive of non-meat eaters in social eating situations was also observed by Kerslake et al. [42] and may represent one of the key roles fulfilled by these products in a way not completely achieved by other alternatives such as nuts and legumes. Overall, the descriptions of how other people affect food choices and behaviour among our participants seem consistent with Cheah et al. [58], who suggested that meat avoidance, by its relation to perceived normative behaviour among socially connected individuals, can be both an opportunity for and a barrier to behavioural change.

Aspects of agency observed here related to wanting to feel like one is “really cooking” when using meat substitutes, and to the importance of having or obtaining the knowledge and skills to do so. This highlights the role of knowledge and skills regarding meat-free meals if consumers’ food choices are to change. People who perceive themselves as able to perform an action are more likely to complete it [59] and evidence also suggests that people are more positive towards objects that they themselves have put effort into making. Sometimes dubbed *the IKEA effect,* this has been demonstrated for flatpack furniture, origami, and Lego creations [60] as well as food preparation [61,62]. This evidence coupled with our observation that participants here responded positively to the design and setup of the study speaks to a role for educating consumers not only in how their food choices affect the environment and their health, but also empowering them with knowledge of the practical aspects of food provisioning and preparation of meat free meals, consistent with previous observations [25,28,44].

### 4.3. Implications and Strategies for Change

Consumers face obstacles in changing their current meat consumption behaviour even when they express a strong intent to do so. Ambivalence was observed among most meat-eating participants here, which may represent both a challenge and an opportunity. Taking advantage of ambivalent people’s heightened sensitivity to information to promote the positive aspects of meat reduction and, critically, its feasibility (e.g., through provision of simple and nutritional meat-free recipes) could reduce that ambivalence and in turn increase the likelihood that these individuals achieve their meat reduction goals. Our participants, overall, held highly positive opinions of the cooking sessions and discussions, and mentioned how useful recipes and cooking guidance for non-meat meals are in general. Empowerment through information, cooking skills, and recipes may be, for this group, conducive to aligning their behaviours with their beliefs rather than vice versa. This would be consistent with evidence showing that the provision of information and recipes was a particularly effective method to reducing meat consumption among “moderate-hindrance” meat-eaters [63], a group comparable to the meat-eating participants here.

Though they were all important factors, the relative importance of animal welfare, health, and environmental motivations on food choices varied across individuals here. Nonetheless, after change is initiated by any of these factors individually, they may create a network of reasons which facilitate maintenance of a low-meat diet, or even lead to eschewing meat entirely. For example, we observed that among non-meat-eating participants here that there tended to be a key issue (typically animal welfare) that initiated dietary change, but that other issues then played a role in maintaining that change. This is also supported by previous work showing that even short-term adherence to changes in meat consumption can lead to sustained reduction, regardless of the initial rationale [64]. Thus, advocating for small changes grounded in only a single strong argument that is salient and powerful for the individual may be paramount, as these may then be maintained and built on over time. Such small changes may also engage the person’s sense of control and agency as they feel more capable of selecting (in public) and preparing (in private) meat-free meals.

Relatedly, the role of meat substitutes in reducing meat consumption also seemed somewhat contingent on consumers feeling comfortable in preparing them and even being able to locate these products in supermarkets. For some individuals, meat substitutes represent convenient transition products, i.e., useful for promoting initial change but not offering a long-term alternative. With time, other meat-free proteins such as legumes may become more enticing, especially if their curiosity for cooking meat-free meals increases. For others, these products may become long-term staples in their diet, allowing them to maintain their now-reduced reliance on meat. Given the strong role of agency and choice identified here and elsewhere, we propose that using digital systems to host cooking classes to empower consumers with knowledge and skills to increase uptake of non-meat proteins would be valuable. This could be explored further by applying a similar design to studies or interventions including cooking in the future. This may also increase accessibility for individuals who may otherwise be restricted in their ability to take part, e.g., due to living rurally.

Although familiarity (branding, sensory, or otherwise) is typically found to affect people’s avoidance or approach of food products, it was not found to be as strong a factor here, possibly because the participants were already familiar with the issues surrounding meat consumption and the existence of meat substitutes. As noted, nearly all participants could name at least one meat substitute product that they had positive views of. We did observe some aversion to ingredients that the participants perceived as unnatural or artificial, consistent with previous research (e.g., Roman et al. [65]). Basing meat substitutes on raw materials that are as familiar to consumers as possible will likely be advantageous. This may be even more effective if the ingredients appeal to consumers’ identity in some way, e.g., oats being perceived as Scandinavian by some participants here improved their view of the products used in the cooking sessions.

### 4.4. Limitations and Strengths

This study is not without limitations. As is often the case with qualitative work, the sample size is relatively small. Although generalisability is not typically the goal of qualitative research, we do aim to extrapolate useful thematic information expected to reflect some aspects of psychological patterns more widely. Since females were over-represented in the sample, this external validity is limited. For other characteristics, such as age and household structure, there was comparably good spread. This study was also somewhat novel in its design, combining home-use and central location setups by hosting digital online cooking sessions. This generates differences in cooking equipment availability and quality, as well as the environment in which the participants were completing the study. Although this arguably improves ecological validity, it could also introduce variance in participants’ experiences that affect the discussions in ways that cannot be reliably controlled.

Despite this, we consider the setup of the study to be a strength as well as a possible limitation. This setup uniquely tackled a previously reported barrier to reducing meat consumption (handling novel products and preparation of tasty meat-free meals) as a component of the design, allowing participants to be specific when discussing practical concerns whilst reinforcing to the meat-eating participants that they can prepare enjoyable plant-based dishes. Complementary psychological and practical aspects of meat substitution were arguably more salient to our participants as a result of this design. This facilitated identifying the strong roles of agency and practical skills in supporting dietary change (rather than characterising these solely as barriers) in a more direct manner than when participants only recall or discuss previous experiences preparing meat-free meals. Taking advantage of technologies such as conference call software to create a virtual central location while keeping participants in the familiar environment of their home has been shown to be a viable approach for both studies where consumers are tasked with preparing meals and focus group studies. Our participants commented that the experience was highly positive and suggested that a similar approach could be taken for cooking classes in general.

## 5. Conclusions

The present work contributes to understanding of attitudes towards meat substitution in meals among individuals who wish to reduce their meat consumption. Overall, our analysis indicates that meat substitutes are useful for facilitating reductions in meat consumption among individuals who have an interest to do so. Meat eating participants, to varying degrees, experienced conflict arising from holding multiple positive and negative beliefs about meat simultaneously (*ambivalence*), justified their own meat-eating behaviour (*rationalisation*), wanted variety and control over their food choices (*agency*), and were sensitive to the views and expectations of other people and the situational context regarding meat (*social and structural* *factors*). The results highlight the need for further research regarding the underlying mechanisms of the meat paradox, in order to find effective communication methods to aid reducing meat consumption among ambivalent consumers. Individual goals and values do not always fit with societal expectations and norms regarding meat. As such, encouraging small changes that may go against the societal grain less but nonetheless can lead to longer-term and wide-spread reductions in meat consumption may be valuable, and contribute to the normalisation of meals without meat. This could be accelerated by efforts to empower consumers with increased knowledge, skills, and confidence in preparing meat-free meals.

## Figures and Tables

**Table 1:** Participant (N = 39) demographics.

	Category	%
Gender	Female	87.2
	Male	12.8
Age Group	18–30	30.8
	31–40	20.5
	41–50	12.8
	51–60	23.1
	61–70	12.8
N adults in household (excl. self) ^1^	0	5.1
	1	38.5
	2+	46.2
	No response	10.3
N children in household ^1^	0	0
	1	28.2
	2+	7.7
	No response	64.1
Vegetarian meal preparation	a. Always (vegetarian/vegan)	10.3
	b. Almost Every Day (5–7 times/week)	15.4
	c. 3–4 times/week	30.8
	d. 1–2 times/week	28.2
	e. 1 time/week	12.8
	f. 1 time/fortnight	2.6

^1^ Optional question.

## Data Availability

The data presented in this study are available on request from the corresponding author.
